# Hidden Relationships between *N*-Glycosylation and Disulfide Bonds in Individual Proteins

**DOI:** 10.3390/ijms23073742

**Published:** 2022-03-29

**Authors:** Tania Bakshi, David Pham, Raminderjeet Kaur, Bingyun Sun

**Affiliations:** 1 Department of Molecular Biology and Biochemistry, Simon Fraser University, Burnaby, BC V5A 1S6, Canada; tania_bakshi@sfu.ca; 2 Department of Computing Science, Simon Fraser University, Burnaby, BC V5A 1S6, Canada; david_pham_2@sfu.ca; 3 Faculty of Health Science, Simon Fraser University, Burnaby, BC V5A 1S6, Canada; rraminde@sfu.ca; 4 Department of Chemistry, Simon Fraser University, Burnaby, BC V5A 1S6, Canada

**Keywords:** posttranslational modifications, *N*-glycosylation, disulfide bonds, membrane and secreted proteins, endoplasmic reticulum quality control, protein structure and function

## Abstract

*N*-Glycosylation (NG) and disulfide bonds (DBs) are two prevalent co/post-translational modifications (PTMs) that are often conserved and coexist in membrane and secreted proteins involved in a large number of diseases. Both in the past and in recent times, the enzymes and chaperones regulating these PTMs have been constantly discovered to directly interact with each other or colocalize in the ER. However, beyond a few model proteins, how such cooperation affects *N*-glycan modification and disulfide bonding at selective sites in individual proteins is largely unknown. Here, we reviewed the literature to discover the current status in understanding the relationships between NG and DBs in individual proteins. Our results showed that more than 2700 human proteins carry both PTMs, and fewer than 2% of them have been investigated in the associations between NG and DBs. We summarized both these proteins with the reported relationships in the two PTMs and the tools used to discover the relationships. We hope that, by exposing this largely understudied field, more investigations can be encouraged to unveil the hidden relationships of NG and DBs in the majority of membranes and secreted proteins for pathophysiological understanding and biotherapeutic development.

## 1. Introduction

Both *N*-glycosylation (NG) and disulfide bonds (DBs) can form co- and post-translational modifications (PTMs) [1] on proteins in the endoplasmic reticulum (ER) while they pass through the secretory pathway. These two modifications are not only common but often evolutionarily conserved in membrane and secreted proteins from prokaryotes to eukaryotes. As two critical modifications, they facilitate protein folding and regulate protein structure, function, stability, and cellular localization. Defects in either one can fail protein ER quality control, trigger unfolded protein response, and cause pathological conditions ranging from heritable congenital disorders of glycosylation as an example to acquired disorders such as cancers, dementia, diabetes, autoimmune, infectious, and cardiovascular diseases [2,3].

Owing to their importance, both NG and DBs have been frequently investigated in numerous proteins. In UniProt [4], over 2700 human proteins have both PTMs annotated. However, the associations between NG and DBs have only been well examined in a few model proteins, such as influenza hemagglutinin (HA) [5,6,7]. Both past and recent discoveries have shown that the enzymes responsible for adding, processing, and degrading *N*-glycans are closely related to proteins with the abilities of adding, isomerizing, and breaking disulfide bonds. Beyond some well-studied model proteins, how NG and DB formation affect each other in the remaining individual proteins is the focus of this review. By reviewing research articles, we generally cataloged the observed relationships from the host proteins into three kinds, including inhibition, promotion, and no relationship. More importantly, we noticed that the majority of the studies did not investigate the interactions between the two PTMs but studied them separately. As a result, some of the observed structural and functional changes in proteins can be a synergistic effect of both instead of one PTM. The reasons are likely that the tools required for these studies were not widely recognized and/or the importance of the associations between them were not commonly appreciated. To raise awareness, this review not only summarizes molecular foundations to support a potentially complex relationship and the current known relationships between NG and DBs in individual proteins but also enlists the relevant tools that can be used for addressing these relationships and provides our recommendations for future high-throughput analysis.

## 2. Molecular Foundations

When nascent proteins are translocated into the ER, NG and DB formation are two closely associated events. NG is a complex process that refers to a dolichol pyrophosphate donor transferring a tetradecasaccharide precursor *en bloc* to the nitrogen atom of the asparagine side chain in a consensus sequence (namely, sequon), i.e., N–X–S/T, in which X can be any amino acid but not proline [8]. Newly added *N*-glycans are further processed while glycosylated proteins fold and mature in the ER. Glycoproteins that fail the quality control in the ER are subjected to the ER-associated degradation process (ERAD) [9]. *N*-Glycans are bulky. The addition of an *N*-glycan can shape the local conformation of a polypeptide chain through the first GlcNAc residue, the triose core, and the outer sugar moieties [10,11,12]. Either intra- or extramolecular *N*-glycans can stabilize a loose polypeptide conformation via both hydrophilic and hydrophobic interactions with the peptide backbone [13]. The stabilized conformation can bring two free cysteine side-chains in close proximity to promote an otherwise slow or energetically costly DB formation, as experimentally proven in model peptides and whole proteins [12,13,14,15].

The covalent disulfide bond is formed by oxidizing two free thiol groups in two cysteine residues in the vicinity. In the ER, DBs can be formed, isomerized, or broken by a number of oxidoreductases. Over 20 ER resident oxidoreductases, also called protein disulfide isomerase (PDI) family enzymes, have been discovered [16]. The characteristic feature of PDI family proteins is the thioredoxin fold, C–X–X–C. This motif is usually the catalytic domain of oxidoreductases. These enzymes can use the cysteines in the motif to exchange electrons in the thiols of the substrate to either form or break DBs [17]. In addition, the mixed DBs formed between the oxidoreductase and the substrate through the thioredoxin fold can often stabilize the substrate and allow further modifications, such as *N*-glycosylation, by other cooperating enzymes in the same complex or nearby [18]. Overall, PDIs can carry out a number of functions, including oxidoreduction, isomerization, and chaperone activity [19].

Due to the spatial requirement of DBs, the protein structure can be largely perturbed or stabilized after the formation of novel DBs or the reshuffling of existing DBs. For glycoproteins carrying bulky *N*-glycans to fold and mature in the ER, the formation of DBs can block or restrain the access of glycoenzymes or chaperones to *N*-glycans. Interestingly, glycoenzymes and glycochaperones are often accompanied by oxidoreductases in the ER, or the glycoenzyme itself has a thioredoxin fold that can interact with thiols in substrates, as recently reviewed by Patel et al. [9]. For example, the oxidoreductases of MAGT1 or TUSC3 are part of STT3B of OST, the enzyme responsive to adding N-glycans to proteins; the oxidoreductases of ERp57 and ERp72 form complexes with CNX/CRT, the ER chaperones of *N*-glycoproteins; in the ER *N*-glycosylation quality control system, UGGT1 itself has thioredoxin-like motifs and interacts with SELENOF (Sep15), a selenocysteine-containing oxidoreductase [20]; further along the thread in the ERAD, BIP binds PDI, P5, and ERdj5, whereas EDEMs bind TXNDC11, P4HB, ERp46, etc. The intriguing roles of NG and DBs are just beginning to be realized.

Beyond the ER, both NG and DBs can be further edited. For example, after exiting the ER, *N*-glycans can be processed in the Golgi [21]. For DBs, a number of enzymes both inside and outside of cells can break and reform these covalent bonds, which often function as switches to activate or suppress host protein functions [22]. How NG and DBs relate to each other beyond the ER remains a question [9]. A complete elucidation of these interactions requires kinetic information of the modification in the time domain, as well as spatial information on their cellular localization. As introduced later, kinetic measurements of PTMs can be performed through traditional pulse-chase or mass spectrometry (MS). The former has been well developed, while the latter is emerging [23]. Spatial information can be delineated through inhibitors specific to an enzymatic step or stressors to a particular organelle.

Nevertheless, not all DBs are linked to every NG in a protein. The functions of both NG and DBs are pleiotropic. The Weerapana group has cataloged the functions of reactive cysteines into five types, including structural, regulatory, redox catalytic, catalytic nucleophile, and metal-binding cysteines [24]. Similarly, the functions of NG range from facilitating local folding to promoting secondary structures, increasing protein stability, protecting hydrophobic surfaces, reducing aggregation, guiding trafficking, and functioning as epitopes for molecular recognition [25].

Given the close molecular associations between enzymes functioning in the formation and processing of the two PTMs in the ER, as well as the broad availability of both PTMs in membrane and secreted proteins, we asked what the current observed relationships of these two PTMs are in individual proteins. Given that one-third of the mammalian proteome is produced through the secretory pathway [26], most proteins have annotated DBs and NG, as shown in UniProt. We anticipated that rich information can be obtained from such exercises. Below, we summarize our findings on both the relationships in individual proteins and the tools used to study them.

## 3. Overview of the Studies

To obtain the relevant articles, we searched PubMed using various keywords, including “*N*-glycosylation and disulfide bonds”, “*N*-linked glycosylation and disulfide bonds”, “*N*-linked glycans and disulfide bonds”, “carbohydrate and disulfide bond”, or “glycans and disulfide”. Furthermore, we included the associated studies cited in the selected papers. The combined list had nearly 600 articles.

**Figure 1:**
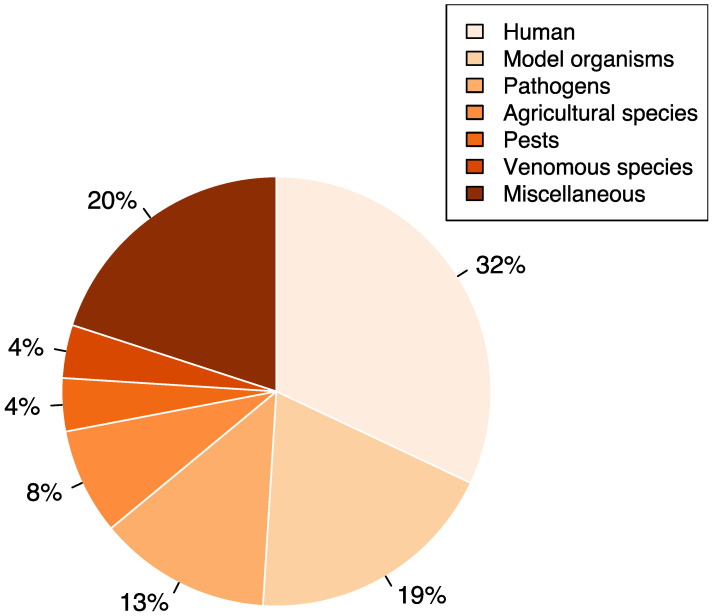
Distribution of species hosting proteins studied for *N*-glycosylation and disulfide bonds.

After establishing the list, we first examined the types of proteins that were studied. Most studied proteins were membrane and secreted proteins of humans, model organisms, and viruses, as shown in Figure 1. Others examined proteins from venomous species such as snakes and wasps, parasites such as hookworms, and agricultural species such as vegetables, fishes, and silkworms. Most of these proteins were surface receptors, adhesion molecules, secreted or membrane-tethered enzymes, cytokines, hormones, signaling molecules, and extracellular matrix proteins. For viruses, the most studied proteins were surface glycoproteins involved in recognition and fusion with host cells. All these results suggested the importance of these two PTMs in diseases originating from cell–protein interactions and cell–cell communications.

**Figure 2:**
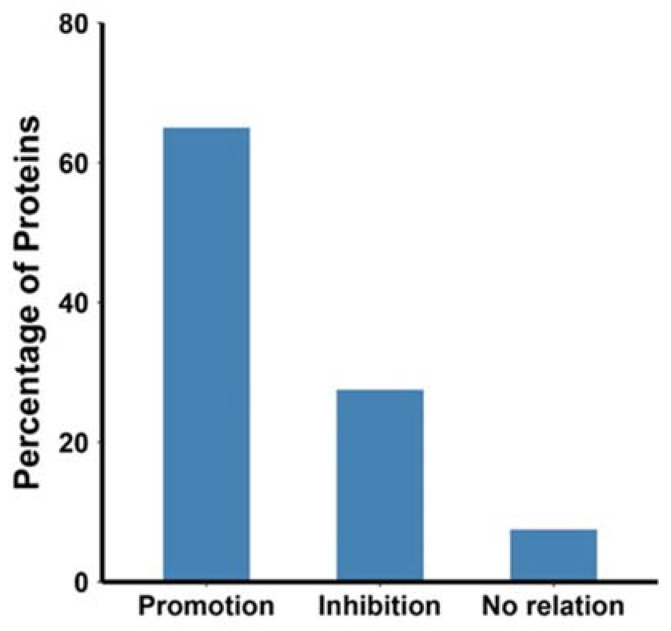
Distribution of different relationships between the *N*-glycosylation and disulfide bonds in the literature examined.

Second, we broadly classified all the reports into four categories. For most studies that included both PTMs but ignored their relationships, we cataloged them as “no studies”. We further divided the investigated relationships into three categories: promotion, inhibition, and independent relationships, as summarized in Figure 2 and detailed in Table 1. In Figure 2, the promoting relationship dominated current studies. This relationship, however, was often observed passively by the diminished or inhibited formation of one type of PTM when the other type of PTM was experimentally removed. To a much lesser extent, an inhibitory relationship was observed, in which the formation of one PTM blocked the development of the other. Only in very limited reports did proteins show independent modifications of these two PTMs, in which the removal of one PTM did not affect the formation or the processing of the other. In the four sections below, we use detailed examples to illustrate these observed relationships.

**Table 1:** Summary of the proteins showing interactions between *N*-glycosylation and disulfide bonds.

UniProt Accession	Protein Name	Position of DBs	Position of *N*-Glycans	Class	Reference
A0A346IHA8	CD4-binding region of the envelope glycoproteins	50–70 ^a^, 219–248 ^a^, 229–240 ^a^, 369–402 ^a^, 362–429, 597–603 ^a^	390, 447	No relation	[27]
Q9Y6R1	SLC4 Na^+^-coupled transporter (NBCe1-A)	583–585, 630–642 ^c^	597, 617	Inhibition	[28]
Q99062	Granulocyte colony-stimulating factor receptor	107–118, 153–162, 224–271, 364–371	27, 69, 104, 110, 365, 450, 547, 555, 586	Promotion	[29]
Q62635	Gastric mucin (rat)		160 ^a^, 420 ^a^, 667 ^a^, 767 ^a^, 837 ^a^, 892 ^a^, 1136 ^a^, 1151 ^a^, 1212 ^a^, 1227 ^a^, 1243 ^a^, 1350 ^a^,	Promotion	[30]
P49018	GPI-anchor transamidase (GPI8) yeast	85 (interchain with C-194 in GPI16) ^b^	256 ^a^, 346 ^a^	Promotion	[31]
P00750	Tissue-type plasminogen activator (t-PA)	41–71, 69–78, 86–97, 91–108, 110–119, 127–208 ^b^, 148–190 ^b^, 179–203 ^b^, 215–296, 236–278, 267–291, 299–430, 342–358 ^b^, 350–419 ^b^, 444–519 ^b^, 476–492 ^b^, 509–537 ^b^	117, 184, 448	Inhibition	[32,33]
P04275	Von Willebrand factor (VWF)	35–162 ^d^, 57–200 ^d^, 388–524 ^d^, 410–559 ^d^, 432–440 ^d^, 509–695, 767–808, 776–804, 810–821, 867–996 ^d^, 889–1031 ^d^, 898–993 ^d^, 914–921 ^d^, 1060–1084, 1071–1111, 1089–1091, 1126–1130, 1149–1169, 1153–1165, 1196–1199, 1234–1237, 1272–1458, 1669–1670, 1686–1872, 1879–1904, 1899–1940 ^d^, 1927–2088, 1950–2085 ^d^, 1972–2123 ^d^, 1993–2001 ^d^, 2724–2774 ^b^, 2739–2788 ^b^, 2750–2804 ^b^, 2754–2806 ^b^	99 ^a^, 156 ^a^, 211 ^a^, 666 ^a^, 857 ^e^, 1147 (atypical) ^e^, 1231 ^e^, 1515, 1574 ^e^, 2223 ^e^, 2290 ^e^, 2357 ^e^, 2400 ^e^, 2546, 2585 ^e^, 2790 ^e^	Promotion	[34,35,36]
P0DN86	Human chorionic gonadotropin beta-subunit	9–57, 23–72, 26–110, 34–88, 38–90, 93–100	13, 30	Promotion	[37,38,39]
	Engineered heterodimeric knob-into-hole Fc fragments	349–354 (interchain)	297	Promotion	[40]
P32906	Yeast-alpha1,2 mannosidase	340–385, 468–471	96, 155, 224	Promotion	[41]
O60896	Receptor activity-modifying protein 3 (RAMP3)	40–72 ^b^, 57–104 ^b^	28, 57, 70, 102	Inhibition	[42]
Q13087	PDIA2	18 (interchain)	127, 284, 516	Inhibition	[43]
P04853	Hemagglutinin-neuraminidase (Sendai virus)	129 (interchain) ^a^	77, 499, 511	Promotion	[21,44]
Q91UL0	Hemagglutinin-neuraminidase (NDV)	123 (interchain), 172–196, 186–247, 238–251, 344–461, 455–465, 531–542	119, 341, 433, 481	Inhibition	[45,46]
	Hemagglutinin (Influenza A virus)	14–466, 52–277, 64–76, 97–139, 281–305, 473–477	8, 22, 38, 81, 165, 285, 483	Promotion	[47,48]
P01229	Human lutropin subunit beta	29–77 ^b^, 43–92 ^b^, 46–130 ^b^, 54–108 ^b^, 58–110 ^b^, 113–120 ^b^	30	Promotion	[49]
P35625	Metalloproteinase inhibitor 3 (TIMP3)	24–91, 26–118, 36–143, 145–192 ^d^, 150–155 ^d^, 163–184 ^d^		Inhibition	[50]
Q16820	Meprin A	103–255, 124–144, 265–427, 273 ^a^ (interchain), 305 (interchain), 492 (interchain) ^d^, 608–619 ^d^, 613–628 ^d^, 630–643 ^d^	41, 152, 234, 270, 330, 426, 452, 546, 553	Promotion	[51,52]
P01130	Low-density lipoprotein (LDL) receptor	27–39 ^e^, 34–52 ^e^, 46–63 ^e^, 68–82 ^e^, 75–95 ^e^, 89–104 ^e^, 109–121 ^b^, 116–134 ^e^, 128–143 ^e^, 148–160 ^e^, 155–173 ^e^, 167–184 ^e^, 197–209 ^e^, 204–222 ^e^, 216–231 ^e^, 236–248 ^e^, 243–261 ^e^, 255–270 ^e^, 276–289 ^e^, 284–302 ^e^, 296–313 ^e^, 318–329 ^e^, 325–338 ^e^, 340–352 ^e^, 358–368 ^e^, 364–377 ^e^, 379–392 ^e^, 667–681 ^e^, 677–696 ^e^, 698–711 ^e^	97 ^a^, 156, 272, 515 ^a^, 657	Inhibition	[53,54,55]
Q02817	MUC2 mucin	59–67 ^b^, 37–169 ^d^, 59–206 ^d^, 391–528 ^d^, 413–563 ^d^, 435–443 ^d^, 860–992 ^d^, 882–1027 ^d^, 891–989 ^d^, 909–916 ^d^, 4481–4622 ^d^, 4503–4661 ^d^, 4527–4535 ^d^, 5075–5122 ^b^, 5089–5136 ^b^, 5098–5152 ^b^, 5102–5154 ^b^	163 ^a^, 423 ^a^, 670 ^a^, 770 ^a^, 894 ^a^, 1139 ^a^, 1154 ^a^, 1215 ^a^, 1230 ^a^, 1246 ^a^, 1787 ^a^, 1820 ^a^, 4339 ^a^, 4351 ^a^, 4362 ^a^, 4373 ^a^, 4422 ^a^, 4438 ^a^, 4502 ^a^, 4616 ^a^, 4627 ^a^, 4752 ^a^, 4787 ^a^, 4881 ^a^, 4888 ^a^, 4955 ^a^, 4970 ^a^, 5019 ^a^, 5038 ^a^, 5069 ^a^	Promotion	[56,57]
P12476	VP7	82–135, 165–249, 191–244, 196–207	69	Promotion	[22,54]
P04156	Major prion protein	179–214	181, 197	Promotion	[58]
P05026	Sodium/potassium-transporting ATPase subunit beta-1	126–149, 159–175, 213–276	158, 193, 265	Promotion	[59,60]
P05231	Interleukin-6 (IL6)	45–51, 74–84	46	Inhibition	[61,62,63]
H2AM12	Glycoprotein Gc	523–550, 580–589, 591–598, 471–487	493, 686	Promotion	[64]
P22146	1,3-beta-Glucanosyltransferase	74–103, 216–348, 234–265, 370–421, 372–462 ^b^, 379–445 ^b^, 398–403 ^b^	40 ^a^, 57, 95 ^a^, 149 ^a^, 165 ^a^, 253, 283 ^a^, 321 ^a^, 409 ^a^, 495 ^a^	Promotion	[65,66]
P32623	Probable glycosidase CRH2		28, 96 ^a^, 190 ^a^, 196 ^a^, 233 ^a^, 237 ^a^, 261 ^a^, 297 ^a^, 310 ^a^	Promotion	[66]
Q9UMF0	Intercellular adhesion molecule-5 (ICAM5)	55–99, 59–103, 142–198, 249–302 ^d^, 344–383 ^d^, 415–470 ^d^, 498–552 ^d^, 580–645 ^d^, 673–725 ^d^, 769–814 ^d^	54,74,137,195,214,274,316,371,397,582,636,645,762,793,794	Promotion	[67,68]
O75829	Chondromodulin-I	131–193 ^b^, 282–286 ^b^, 283–323 ^b^, 293–317 ^b^, 297–313 ^b^	243 ^a^	No relation	[69]
P40225	Thrombopoietin	7–151, 29–85	197, 206, 234, 255, 340 ^a^, 348 ^a^	No relation	[70,71]
Q9UNQ0	ABCG2 protein	592–608, 603 (interchain)	596	Promotion	[8,72,73]
P56817	beta-Site APP-cleaving enzyme (BACE)	216–420, 278–443, 330–380	153 ^a^, 172 ^a^, 223 ^a^, 354 ^a^	Promotion	[74]
Q9H1U4	Multiple epidermal growth factor-like domains protein 9 (MEGF9)	204–217 ^a^, 206–224 ^a^, 226–235 ^a^, 238–251 ^a^, 254–266 ^a^, 256–272 ^a^, 274–283 ^a^, 286–298 ^a^, 301–310 ^a^, 303–317 ^a^, 320–329 ^a^, 332–346 ^a^, 349–360 ^a^, 351–371 ^a^, 374–383 ^a^, 386–397 ^a^, 400–415 ^a^, 402–422 ^a^, 425–434 ^a^, 437–449 ^a^	40 ^a^, 182 ^a^, 205 ^a^, 218 ^a^, 245 ^a^, 267 ^a^, 305 ^a^, 428 ^a^, 468 ^a^, 481 ^a^, 500 ^a^	Inhibition	[75]
P53634	Cathepsin	30–118, 54–136 ^e^, 255–298 ^e^, 291–331 ^e^, 321–337 ^e^	29	Inhibition	[76,77]
P08709	Coagulation factor VII	348–367	360	Inhibition	[76]
Q07837	rBAT	242–273, 571–666, 673–685	214, 261, 332, 495, 513, 575	Promotion	[78]
O75355	Nucleoside triphosphate diphosphohydrolase 3 (NTPDase3)	92–116, 261–308, 289–334, 347–353, 399–422	81 ^a^, 149 ^a^, 238 ^a^, 381 ^a^, 392 ^a^, 402 ^a^, 454 ^a^	Inhibition	[79]
P08563	E2 glycoprotein (rubella virus)		53, 71, 115	Promotion	[80]
Q9H9S5	Fukutin-related protein (FKRP)	6 (interchain)	172, 209	No relation	[81]
O14773	Tripeptidyl-peptidase I	111–122, 365–526, 522–537	210, 222, 286, 313, 443	Promotion	[82,83,84,85]
P15813	CD1d	120–184, 224–279	20, 42, 108, 163	Promotion	[86,87,88,89]
O14656	TorsinA	44–162 ^a^, 280–319	143, 158	Promotion	[90,91]
P21825	Translocation protein SEC62		153, 62 ^e^	Promotion	[92]
P04070	Protein C	331–345	97, 248, 313, 329 (atypical)	Promotion	[93]
K7WJ21	PtrMAN6	448, 452, 456	23, 194, 227, 375, 392	No relation	[94]
P01848/P01850	TCR alpha and beta	22–72, 94 (with C-130 in TRBC1 or TRBC2) and 30–95, 130 (with C-94 in TRAC)	32, 66, 77 ^d^, 113 ^d^ and 69 ^d^	Inhibition	[95]
P01857	Immunoglobulin G1 Fc	27–83 ^d^, 103 ^e^, 109 ^e^, 112 ^e^, 144–204 ^d^, 250–308 ^d^	180, 297	Promotion	[84,96,97,98]
	Nicotinic acetylcholine receptor fragment	128–142	141	Promotion	[12]
Q07108	CD69	68 (interchain) ^e^, 85–96 ^e^, 113–194 ^e^, 173–186 ^e^	111 (atypical), 166	Inhibition	[99,100]
P15813	Antigen-presenting glycoprotein CD1d	120–184 ^e^, 224–279 ^e^	38 ^e^, 60 ^e^, 126 ^e^, 181 ^e^	Promotion	[101,102]
P04439	MHC class I heavy chain	125–188 ^e^, 227–283 ^e^	110 ^e^	Promotion	[103,104]

^a^—predicted by sequence analysis (accessed from UniProt); ^b^—by similarity (accessed from UniProt); ^c^—a discrepancy between NG sites in the abstract and main text was found in [28]. We used the sites mentioned in the main text as they correspond with UniProt; ^d^—PROSITE-ProRule annotation; ^e^—UniProt.

## 4. Promoting Relationship

**Figure 3:**
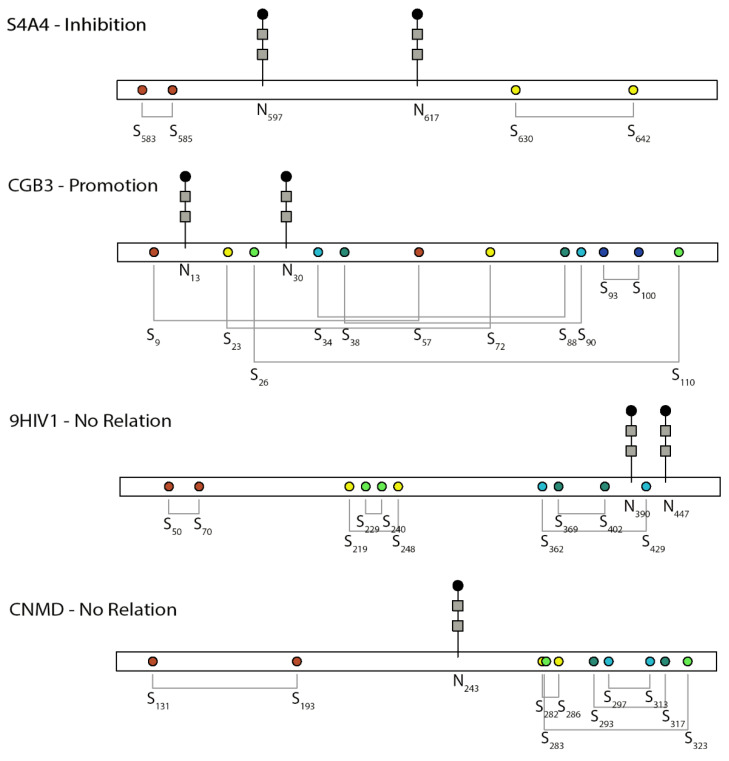
Schematic of the relative position between *N*-glycosylation and disulfide bonds and their relationship in the selected proteins discussed here. S4A4, UniProt ID of human SLC4 Na^+^-coupled transporter, NBCe1; CGB3, UniProt ID of human chorionic gonadotropin beta-subunit; 9HIV1, UniProt ID of human immunodeficiency virus envelope glycoprotein; CNMD, UniProt ID of human chondromodulin-1. Colors indicate different disulfide bond pairs. The *N*-glycan sign does not represent the actual sugar structure.

Feng et al. [37] demonstrated through kinetic studies of intracellular folding of the human chorionic gonadotropin (hCG)-β subunit that NG facilitated the rapid formation of DBs and the folding of the hCG-β subunit, which harbors six DBs [39]. The relative positions of NG and DBs are shown in Figure 3. Lacking the two NG sites slowed down the folding of the β subunit more than fourfold from 7 min to 33 min in CHO cells, and the slow formation of DBs retained the misfolded proteins up to 5 h in the ER before degradation [37]. The co-expression of the α subunit could assist the appropriate folding and secretion of the β subunit of the hormone lacking the NG. Among the six DBs in the hCG-β subunit shown in Figure 3, the formation of Cys^34^–Cys^88^ occurred earlier than that of Cys^9^–Cys^57^/Cys^38^–Cys^90^, while the remaining three pairs occurred later [39]. The first three pairs are important in protein folding and secretion and *N*-glycan processing. Eliminating these early formed DBs rendered part of the *N*-glycans to be high mannose instead of complex glycans, which were sensitive to ER quality control and degradation.

Another study was conducted in the β subunit of Na, K-ATPase. Na, K-ATPase is a plasma membrane transporter that is responsible for the maintenance of potassium and sodium homeostasis in animal cells [59]. The functional β subunit is a type II glycoprotein composed of a large C-terminal ectodomain with three NG sites (Asn^158^, Asn^193^, Asn^265^) and three conserved DBs (Cys^126^–Cys^149^, Cys^159^–Cys^175^, and Cys^213^–Cys^276^) [59]. The mutation of Cys^126^–Cys^149^ increased the non-glycosylated proportion of the protein compared to the wildtype from the Western blot [60], suggesting a promoting relationship. Mutating each of the three glycosylation sites indicated their involvement in initial folding [59]. The acquisition of at least one sugar moiety was necessary for the β subunit to ensure its association with the α subunit through pulse chase. Interestingly, when all three *N*-glycans were removed, the protein did not form aggregates through DBs but permanently associated with BIP from degradation [59].

In addition to hCG and Na, K-ATPase, multiple other examples also indicate a “strengthening” relationship between NG and DB. Mirazimi and Svensson [105] showed that the chief role of NG on rotavirus VP7 is to facilitate correct intermolecular DB formation in dimerization. Removal of NG induced VP7 misfolding through random intermolecular DBs. Similar effects were also observed for MUC2 [56], vWF [36], meprin A [52], and hemagglutinin [47], as shown in Table 1.

## 5. Inhibitory Relationship

The hemagglutinin-neuraminidase (HN) glycoprotein of Newcastle disease virus (NDV) is responsible for virus attachment to host cell receptors, thereby initiating infection [4]. The HN protein is a type II membrane protein containing six potential NG sites: Asn^119^, Asn^341^, Asn^433^, Asn^481^, Asn^508^, and Asn^538^ [45]. Among them, only four (Asn^119^, Asn^341^, Asn^433^, Asn^481^) are utilized for NG [106]. The protein also has 13 cysteine residues in the ectodomain [106], as summarized in Table 1. The cysteine residue closest to the membrane anchor (Cys^123^) is involved in an intermolecular DB [107,108], while the other 12 cysteine residues form intramolecular DBs [107].

McGinnes and Morrison found that intramolecular DBs might play a critical role in the usage of glycosylation sites [107]. They explored whether DB formation could be a determinant of the two unused glycosylation sites, Asn^508^ (site 5) and Asn^538^ (site 6), in HN protein [45]. Removing Cys^531^–Cys^542^ flanking the unused glycosylation site Asn^538^ by mutation or DTT promoted the NG of Asn^538^ for an efficiency of 39–59% and 26–27%, respectively [45]. The successful NG was supported by the deglycosylation analysis with endo H. Under similar conditions, the usage of the non-glycosylated site 5, Asn^508^, which is far from any DB, was not improved. Together, these results suggest that the glycosylation of Asn^538^ is under steric hindrance by the DB in the vicinity [45], whereas the non-glycosylated Asn^508^ could be caused by other factors not related to DBs [45].

Another study investigating the lack of sequon utilization in tissue-type plasminogen activator (t-PA) reported similar findings that folding and DB formation of t-PA negatively impact the extent of core *N*-glycosylation [33]. As a result, they suggested that variable usage of glycosylation sites could be caused by the transient accessibility and appropriate orientation of the sequon relative to the transferase or dolichol-linked donor in a folding event [33].

Human sodium bicarbonate cotransporter 1, NBCe1 (*SLC4A4* gene), is an electrogenic sodium/bicarbonate cotransporter localized in the plasma membrane [4]. The malfunction of this gene is related to a series of diseases in the kidney, eye, ear, brain, and tooth. All SLC4 Na^+^-coupled transporters are multipass transmembrane proteins containing a large extracellular loop (EL-3) with multiple NG consensus sites and four highly conserved cysteines [109]. NBCe1-A, one of the three variants, is a homodimer, and its two EL-3 loops form unique conformations that are potentially critical to the function of the protein [28]. In the EL-3 loop of NBCe1-A, two sequons are glycosylated (Asn^597^ and Asn^617^) but not Asn^592^, and four conserved cysteines form two intramolecular DBs (Cys^583^–Cys^585^ and Cys^617^–Cys^642^), as shown in Figure 3 [28].

In a detailed study evaluating the interplay between DBs and NG to define the EL-3 loop topology in NBCe1, it was found that the two EL-3 loops of the dimer formed a unique clove conformation [28]. This conformation was “finely tuned” by glycosylation [28]. In the absence of Cys^583^–Cys^585^ or the two NG sites, the third NG site, Asn^592^, became glycosylated. With glycans, the two DBs were deeply buried from the external surface of the EL-3 loop, which can sustain DDT-induced denaturation and enzymatic digestion under basic conditions. Losing both DBs and NG made the loop adopt an extended structure that could not be recognized by the designated antibody and was susceptible to chymotrypsin digestion [28]. Instead of considering the steric hindrance between DBs and NG at Asn^592^, the authors hypothesized that Asn^592^ was originally glycosylated at the nascent polypeptide chain of NBCe1-A and later removed when DBs and other glycosylation sites were formed [28]. Removing NG did not affect the formation of two DBs; however, an additional removal of one cysteine in the DBs by mutagenesis promoted the formation of intermolecular DBs in the homodimer, which maintained transport function [28]. A complex relationship must exist between NG and DBs in determining the final folding of EL-3 during NBCe1-A protein maturation; however, no kinetic experiments were performed to monitor protein folding, and ER maturation was not specifically probed. It is, therefore, difficult to elucidate how ER resident enzymes facilitate these processes. The study, on the other hand, had systematic structural delineation by a combinatorial mutation of all four cysteines in two DBs for a total of 12 mutants.

## 6. Independent Relationship

Envelope glycoprotein 160 (gp160) on human immunodeficiency virus (HIV) is critical for viral binding to the CD4 receptor and fusion with CD4^+^ cells. The precursor gp160 needs to be cleaved to gp120 and gp41 to activate the binding domain on gp120 with CD4. A study on the linkage region between gp120 and gp41, which is also the future binding site of gp120 to CD4, suggested that DBs and NG in this region function independently [27]. The relative position between NG and DBs in this region is shown in Figure 3. Cys^402^ and Cys^429^ are both located in the linker region but form separate DBs, of which Cys^402^ is critical for cleavage. Mutation of Cys^402^ not only prevented the cleavage but also affected the transport of gp160 and the future binding of gp120 to CD4^+^ cells [110,111]. Around Cys^402^, there are two occupied NG sites Asn^390^ and Asn^447^. Mutating these NG sites did not affect disulfide bonding through Cys^402^ or the relevant functions, suggesting an independent relationship between DBs and NG.

In another study of the 25 kD extracellular matrix protein chondromodulin-I (ChM-I), NG was critical in its solubility but had no effect on DBs [69]. ChM-I is a secreted protein and has two separate domains, in which the hydrophilic N-terminal domain is heavily glycosylated by one *N*-glycan and two *O*-glycans, whereas the hydrophobic C-terminal domain harbors four DBs. As the two domains are separated, the removal of either the NG or the N-terminal domain seems to have no effect on the formation of DBs in the C-terminal domain, as shown in Figure 3.

## 7. Unknown Relation

For most of our searched studies that concerned both DBs and NG, the exact relationship between the two PTMs was not experimentally examined. Half of these studies focused on experimental mapping of their sites without functional studies. One-third of the remaining studies only predicted the potential DBs and NG by sequence alignment or computational modeling without experimental data. For the articles that did examine the functions of both modifications, many of them did not study or discuss their interactions but rather examined them separately. For a very small number of papers, the potential interactions were hypothesized but not experimentally verified.

For example, a very nice study investigated the role of NG and DB in the rat G protein-coupled receptor class C, group 6, member A (GPRC6A) [112], a widely expressed GPCR that functions importantly in many diseases ranging from metabolic syndrome to cancer [113,114]. This protein is a class C GPCR with a large N-terminal extracellular domain (ECD), which contains a Venus-flytrap (VFT) domain and a cysteine-rich domain (CRD) [4]. The VFT domain is for ligand binding, and the CRD domain is for signal transfer [112].

It was observed that the ECD domain of GPRC6A consists of nine sequons [112]. Only seven asparagine residues carry *N*-glycans. Five of them are in the VFT domain, and Asn^555^ and Asn^567^ are located in the CRD [112]. The VFT domain also has two conserved cysteines (Cys^122^ and Cys^131^), whereas the CRD domain has nine conserved cysteines, eight of which form intra-CRD DBs [113]. Through analysis of different mutants by SDS-PAGE, it was found that Asn^555^ is important for protein surface expression and that Asn^567^ regulates receptor function. Furthermore, from the studies of two cysteines, Cys^122^ and Cys^131^, C^131^ contributed to the formation of a homodimer through an intermolecular disulfide bridge [112], and Cys^122^ contributed to the interdomain DB between VFT and CRD [115]. Mutation of C131A abolished the intermolecular DB and homodimer formation but did not impair receptor surface expression and its function, whereas mutation of C122A was responsible for the lowered signal response (40%) and higher (50%) surface expression [112]. This result suggested that the C122A mutation causes certain conformational changes. Not only is Cys^122^ next to Asn^121^, but the DB between CRD and VFT domains can also be largely shaped by the seven *N*-glycans carried by these two domains. It is likely that NG plays a role in the potential conformation changes or intermolecular DB formation; however, no experiments or discussion were presented on the relationships between NG and cysteine disulfide bridges or cysteines in the paper.

Another study explored the role of the conserved cysteines and NG sites among all alphaherpesviruses such as herpes simplex virus 1 (HSV-1) in virus production and membrane fusion by single- and double-site directed mutagenesis [116]. Glycoprotein K (gK) is a conserved virion protein in all alphaherpesviruses [117]. The N-terminal extracellular domain of gK is important for HSV-1 to enter neurons via axonal termini. This domain contains two conserved NG sites at Asn^48^ and Asn^58^ and four conserved cysteines for two potential disulfide pairs of Cys^37^–Cys^114^ and Cys^82^–Cys^243^ according to single-cysteine mutation and computational modeling [116].

It was found that viruses lacking Asn^58^ or lacking both sites (Asn^48^, Asn^58^) had enhanced fusion [116]. Interestingly, deletion of Cys^37^ or Cys^114^ led to a gK-null phenotype of few plaques, whereas mutation of Cys^82^ or Cys^243^ caused enhanced cell fusion. The authors provided an extensive discussion on the potential interactions between NG and DB on the basis of the known studies and hypothesized that the removal of NG at Asn^58^ could displace the DB formation, as the deletion of the Cys^82^–Cys^243^ disulfide recapitulated a similar fusion phenotype of Asn58A. However, the authors did not further verify this hypothesis, such as examining the presence of DBs through gel shift assays, labeling assays for free thiols, or MS characterization. Therefore, the authors in the end did not entail the specific relationship more than stating the presence of “a potentially important relationship” [116].

Related to the ongoing COVID-19 pandemic, the immunogen SARS-CoV-2 spike protein and its endogenous binding target ACE2 are both heavily glycosylated with numerous DBs. The SARS-CoV-2 S protein has a total of 22 sequons that to various extents are all glycosylated [118,119,120,121,122,123,124,125], seven *O*-glycosylations [118,125], and 40 cysteines with 15 DBs [118]. Similarly, ACE2 was mapped to have seven NG sites [36,125], one *O*-glycosylation [125], and four DBs [126,127]. According to mutations, molecular dynamics simulations of protein structures, and sequence alignment studies, eliminating certain DBs or NG on both ACE2 and SARS-CoV-2 S proteins can alter binding affinity to each other and change virus infectivity. For example, Cys^480^–Cys^488^ is considered the most important pair in the receptor-binding domain (RBD) of SARS-CoV-2 S proteins, and this pair participates in binding to the N-terminal of the host receptor that forms a stable SARS-COV-2 and ACE2 complex [126,127,128]. In addition, Cys^133^–Cys^141^ of ACE2 is responsible for making the loop at dimer interference [76,128] and is predicted to be crucial for making interactions with the spike protein of SARS-CoV-2 [127]. Deletion of Asn^90^ glycosylation of ACE2 increased the binding to S proteins, while removal of Asn^322^ of ACE2 decreased virus binding and infection [125,129]. From the perspective of SARS-CoV-2 S protein, the Ser^309^ neutralizing antibody binds Asn^234^ glycosylated RBD [32], and double mutations of N165A/N234A [35] and N331Q/N343Q [53] in S protein both reduced the binding between the immunogen and the receptor. Despite the extensive and rapid studies of NG and DBs in S proteins and ACE2 in the past 2 years, no studies have examined the relationship between DBs and NG in these two proteins. This phenomenon clearly indicates the severe understudy in this important field.

## 8. Common Methods

Successful studies of the relationships between DB and NG relied on suitable tools. Table 2 and Table 3 summarize common methods used in studies of NG and DB, respectively.

**Table 2:** Summary of methodologies for the identification and structural analysis of *N*-glycosylation in proteins.

**NG detection**	Staining procedures [130]	
	Resolve protein on SDS-PAGE and stain the gel for glycoproteins	
	Affinity-based methods [130]	
	Saccharide-binding protein (lectin-based) Enzyme-based methods Antibody-based methods	
	NMR spectroscopy [131,132]	
	X-ray crystallography [133]	
	Circular dichroism (CD) spectroscopy [64]	
**NG structural analysis**	Requires *N*-glycan removal prior to further analysis which can be achieved by	Chromatography
	Enzymatic removal [130,134] PNGase F Endo-H and peptide: *N*-glycanase [67] Chemical removal [130] β-Elimination Alkaline borohydride [135] Hydrazinolysis	Weak anion exchange (WAX) [136] Gel filtration High-performance anion-exchange chromatography with pulsed amperometric detection (HPAEC-PAD) [137] Normal-phase high-performance liquid chromatography (NP-HPLC) [138] Reverse-phase HPLC (RP-HPLC) [139] Mass spectrometry [140,141,142,143,144,145,146] MALDI-MS, ESI-MS, or LC–ESI-MS [147] LC–MS/MS [148] Targeted MS/MS [149]
**NG functional analysis**	Chemical tools: inhibitors of glycosyltransferases and glycosidases (in vivo) [150]	
	Tunicamycin Plant alkaloids: australine, castanospermine [151], deoxynojirimycin [151], deoxymannojirimycin, kifunensine, swainsonine, and mannostatin A	
	Physical tools: adjust temperature, ATP, pH, etc.	
	Site-directed mutagenesis	
	Genetic glyco-engineering [152]	
	Involves introduction of heterologous glycosylation machinery or inactivation of endogenous enzymes.	

**Table 3:** Summary of the methodologies for the detection and analysis of disulfide bonds in proteins.

**Quantify the amount of unbound cysteine residues**	Chemical labeling and spectroscopic detection [153]	
	Ellman assay *N*-(1-pyrenyl)maleimide (NPM) labeling	
**Reduction methods**	Reducing enzymes [153]	
	2-Mercaptoethanol (ME) Dithiothreitol (DTT) Tris(2-carboxyethyl)phosphine (TCEP) Tris(2-hydroxyethyl)phosphine (THP)	
**Detection/Analysis of DB**	Edman degradation sequencing [154]	
	NMR spectroscopy [155,156]	
	X-ray crystallography [157]	
	2-Nitro-5-thiosulfobenzoate (NTSB) assay [158,159]	
	Electrophoretic methods [153,160] Capillary electrophoresis sodium dodecyl sulfate (CE-SDS) Nonreducing SDS polyacrylamide gel electrophoresis (SDS-PAGE) [160,161] Diagonal gel electrophoresis [162]	
	Mass spectrometry approaches: Front-end separation [153,163,164,165,166,167,168,169]	Mass spectrometry approaches: Fragmentation types
	LC–MS/MS LC–ESI-MS/MS ETD-MS/MS ESI-MS/MS MS/MS/MS	Activated ion ETD (AI-ETD) [170,171] CID [172,173] HCD [174] EThcD [175,176] Ultraviolet photodissociation (UVPD)–MS [177]
**Detect structural changes**	Partial proteolysis by enzymes such as trypsin and pepsin	
	Functional assays	
	Epitope tags such as haemagglutinin, hexahistidine, V5, FLAG (visualized using antibodies) Biotin tags	
	Site-directed mutagenesis	

For both modifications, conventional analytical methods include NMR [155,156], X-ray crystallography [157], cryoEM, CD for structure analysis, Edman degradation [154] and mass spectrometry (MS) for sequencing analysis, and electrophoresis for protein migration mobility analysis [153,160]. Gel shift assay by electrophoresis is a readily accessible and effective analytical method widely employed to study both modifications. Electrophoretic methods such as SDS-PAGE and capillary electrophoresis under reducing and nonreducing conditions are simple assays for primary analysis of the overall DBs in a protein. By comparing the differential electrophoretic mobility, researchers can deduce the presence of DBs. In particular, diagonal electrophoresis, which conducts sequential nonreducing and reducing electrophoresis in two orthogonal dimensions of the same sample, allows easy identification of DBs [162]. For NG, enzymes and chemical removal of sugar moieties can be identified by mobility shifts in SDS-PAGE. Due to the size of *N*-glycans, their multiplicity can also be resolved by SDS-PAGE from the corresponding ladder-shaped protein bands. Combined with pulse-chasing using isotopes such as [^35^S]Met, the kinetics of the formation, processing, maturation, and degradation of these modifications are often monitored by the gel shift assay.

X-ray analysis of *N*-glycosylation can be challenging due to the heterogenous glycoforms that impede diffraction-quality crystallization. Nevertheless, the 3D structures of *N*-glycans in glycoproteins have been growing in PDB [178]. Various methods have been explored, including engineering the host cell glycosylation machinery to produce homogenous *N*-glycan-modified proteins for X-ray crystallography [133]. Many NMR methods have been developed to study the structure of *N*-glycans, intact *N*-glycoproteins, *N*-glycoprotein complexes, and model *N*-glycosylated peptides [131,132]. Synthetic model glycopeptides have unique advantages in terms of forming well-defined sequences and structures to interrogate their conformational effect in great detail under NMR [132]. In particular, the relations between NG and DBs have been characterized using synthetic model peptides, such as those derived from nicotinic acetylcholine receptor and prion protein [12,58]. In addition, MS has also recently been developed to decipher glycoprotein complex interactions through *N*-glycans, in which various glycoforms can be examined individually [179].

For molecular engineering, mutagenesis is another common method that is widely used to accurately pinpoint the site of modifications and to study the structural and functional consequence upon complete, permanent, and selective removal of some or all of these PTMs. The most frequently used mutagenesis is single-amino-acid substitution, even though deletion of one or a chain of amino acids exists. It is worth mentioning the choice of amino-acid replacements. Commonly, cysteines are mutated to alanines (A) or serines (S), whereas asparagines are replaced by aspartic (D) and glutamic acids (E) or glutamines (Q), even though some studies replaced threonine (T)/serine (S) in the sequons to abolish NG.

Chemical removal or tagging of DBs and NG is also frequently employed to identify their presence and functions. For DBs, reducing agents can be used to disrupt the covalent bond, and thiol-reactive chemical groups can modify the free thiols to distinguish them from those that are occupied by DBs. Either a gel shift assay or MS can be employed downstream to identify changes in these chemical perturbations. Chemical removal of NG can be catalyzed by enzymes. Using enzymatic selectivity, different glycans can be readily distinguished. For example, according to the degree of processing, *N*-glycans have three types, i.e., high mannose, complex, and hybrid *N*-glycans. Endoglycosidase (endo H) cannot cleave complex *N*-glycans, yet *N*-glycosidase F (PNGase F) can [134]. Therefore, the two enzymes are frequently used to delineate the type of *N*-glycans. Chemical removal can also be facilitated by small molecules, such as base-assisted beta-elimination and hydrazinolysis [130], which are less selective than enzymatic reactions.

For structural characterization upon changes by DBs and NG, in addition to the instrumental approaches mentioned above, several biochemical approaches have also been developed. First, enzymes, particularly peptidase/protease, have been used to examine the overall structures of proteins. Both trypsin and pepsin were used to assess the compactness of the protein folding according to digestion efficiency. Second, for specific epitopes of a protein, antibodies were developed for rapid and specific recognition.

In addition to the above in vitro analysis methods, in vivo tools to interrogate the pathways in the formation and processing of the two PTMs were made available. A selective perturbation to the pathways can be achieved through molecularly engineered knockout, knockdown, or knock-in of a particular enzyme or chaperone. Targeted changes can also be elicited through pharmacological inhibition by small molecules. Less selective conditions, such as dithiothreitol (DTT) and 2-mercaptoethanol (2-ME) treatments, were also used to induce a global reduction of all DBs in vivo.

One important aspect of characterizing the relationships between NG and DBs is to examine the kinetics of protein maturation in the secretory pathway, as well as the kinetics of enzymatic reactions that regulate NG and DB formation and processing. Due to the migration shift in the gel after the formation of *N*-glycans and DBs, the protein maturation kinetics have often been examined by pulse-chase gel-shift assays. Regarding the kinetics of glycoenzymes with oxidoreductase activities, available studies are very limited. Historically, radioisotope labeling was used to probe enzymatic reactions in vitro to build predictive models for *N*-glycosylation [180,181]. Later, the MS characterization of glycans with and without stable isotope labeling was adopted for safer measurements [182,183,184]. Recently, targeted MS was developed to quantify the kinetics of stable isotope-labeled glycopeptides for more accurate modeling, in which not only the rates of glycan synthesis/processing but also the amino-acid sequences around glycosylation sites were monitored for “cell, enzyme, and glycosylation” site-specific analysis [23].

Among all the techniques, we would like to highlight MS in the characterization of the two modifications. With the advent of modern instrumentation, the method measures the mass of peptides down to sub-ppm accuracy and attomole sensitivity. The instrument holds promise for sequencing, i.e., structurally resolved, complex biological samples including all proteins, peptides, nucleic acids, carbohydrates, lipids, and metabolites within [151]. It has been playing ever increasingly important roles in deconvoluting the structures of proteins, including their DBs and glycosylation. In recent years, the application of the technique has been moved from studying one protein at a time to studying a sub-proteome by enrichment through a native moiety or chemical tagging. For example, glycoproteins and glycopeptides can be enriched by lectins or hydrophilic metals, charcoal, organic sorbents, or chemical bonding to sorbents through hydrazone and boronic acid diesters and subsequently analyzed by MS for identification, quantification, or structural interpretation [185,186,187]. Similarly, the thiol group has been a conventional substrate for MS analysis either at the individual protein level or at the proteome level using methods such as ICAT [188]. With both label- and label-free quantitation rapidly developing, MS characterization holds the potential both for steady-state analyses and for kinetic interrogations in modeling biological processes. Several reviews are available that summarize the field of MS characterization in either DBs [166,188] or NG [185,186,187].

## 9. Final Remarks

It is widely accepted that both NG and BDs can affect the folding, maturation, trafficking, and degradation of host proteins; however, how to effectively control and engineer these PTMs in individual proteins for disease prevention and treatments, respectively, remains in its infancy. Unlike existing reviews on the mechanisms of enzymes functioning in NG and DB formation pathways, our study focused on the relationships of the two PTMs discovered in individual proteins. After reviewing more than 500 papers that investigated both modifications in one protein, we noticed that most studies only mapped their positions or studied their functions separately. Fewer than 100 articles have experimentally addressed the relationships between the two PTMs. We summarized the studied proteins in Table 1.

From the intriguing cooperation observed between *N*-glycoenzymes and oxidoreductases in the ER, it is envisaged that close relationships between NG and DBs are anticipated to widely exist in membrane and secreted proteins. Compared to the total human proteins annotated in UniProt with DBs and NGs, the proteins in Table 1 comprise less than 2%. As a result, for the most studied STT3B and STT3A substrate preferences, their complete responsive sites in all substrate proteins are still elusive. A recent discovery-based study carried out by proteomics on STT3A and STT3B substrate pools uncovered some interesting proteins [75] that could not be explained by the known mechanisms. During CNX/CRT-chaperoned *N*-glycoprotein folding, oxidoreductases are involved. It is known that there are ERp57 obligate and ERp57 facultative substrates [189]. ERp72 was discovered to be the alternative enzyme that acts on facultative substrates in the absence of ERp57; however, it is unclear how the obligate and facultative substrates are determined in vivo. For other complexes, such as EDEMs in ERAD, their protein substrates are just starting to emerge.

**Figure 4:**
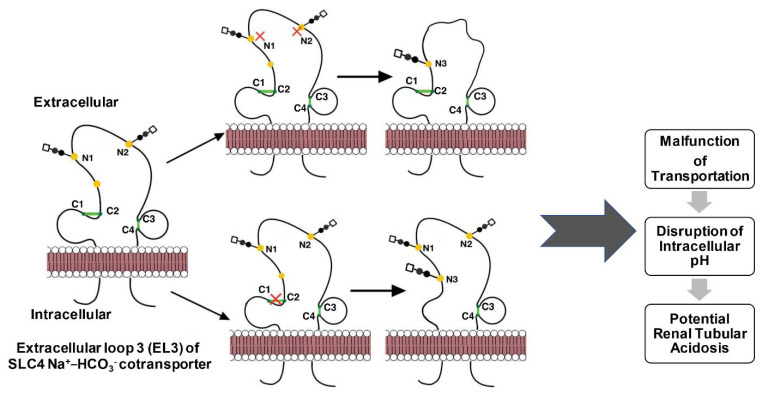
Molecular pathophysiology of extracellular loop 3 of NBCe1-A caused by mutual relationships between *N*-glycosylation and disulfide bonds.

Even though relatively few proteins have been studied on the mutual relations between NG and DBs as exemplified in Figure 4, they are important disease biomarkers and therapeutic targets. Understanding the function and regulation of these PTMs is, therefore, critically important in disease treatment and prevention. Most of the studies used mutagenesis to remove one or both PTMs for structural and functional effects; however, a few studies introduced novel PTMs into proteins to engineer protein-based drugs/vaccines for better stability and efficacy [48,190,191]. In addition, knowledge obtained from studying these modifications can help researchers gain insights into early disease diagnosis and prevention [50,58].

Due to the diverse structures possessed by individual proteins and the complex relationships, it is important to examine the entire sub-proteome to gain comprehensive knowledge. The current intriguing interactions between the two PTMs derived from model proteins should serve as an encouraging start for an exciting field. We believe that the MS-led new generation of high-throughput high-accuracy analyses can quickly move this field forward. Lastly, we hope that this review will encourage future studies to investigate the relationship between NG and DBs and to better disclose their hidden linkages in the remaining 98% of the proteins for novel insights into their structural and functional roles.

## Data Availability

Not applicable.
